# Lead-Free FACsSnI_3_ Based Perovskite Solar Cell: Designing Hole and Electron Transport Layer

**DOI:** 10.3390/nano13091524

**Published:** 2023-04-30

**Authors:** Syed Abdul Moiz, Ahmed N. M. Alahmadi, Mohammed Saleh Alshaikh

**Affiliations:** Device Simulation Laboratory, Department of Electrical Engineering, College of Engineering and Islamic Architecture, Umm Al-Qura University, Makkah 21955, Saudi Arabia; anmahmadi@uqu.edu.sa (A.N.M.A.); msshaikh@uqu.edu.sa (M.S.A.)

**Keywords:** perovskite solar cell, lead-free perovskite, FA_0.85_Cs_0.15_SnI_3_, SCAPS-1D

## Abstract

In recent years, lead-based perovskites solar cells have demonstrated excellent power-conversion efficiency. Despite their remarkable progress, the commercialization of lead-based perovskites is hampered by lead toxicity concerns. The recently discovered non-toxic FACsSnI_3_ perovskite has the potential to replace lead-based perovskites in solar cell applications. Since the perovskite material FACsSnI_3_ (FA_0.85_Cs_0.15_SnI_3_) is relatively new, there is a lack of information, particularly regarding the design features required for electron and hole-transport layers for efficient photovoltaic responses. The important variables, such as electron affinity, energy band gap, film thickness, and doping density of both electron and hole-transport layers, were simulated and modeled separately and iteratively in this study to achieve the most efficient photovoltaic response. Finally, the absorber layer thickness of FACsSnI_3_ perovskite is tuned to achieve a maximum power-conversion efficiency of slightly more than 24%. We hope that the findings of this study will serve as a strong guideline for future research and the design of lead-free perovskite solar cells for efficient photovoltaic responses.

## 1. Introduction

In recent years, tremendous advances in the power-conversion efficiency of lead (Pb)-based perovskite solar cells have been recorded, indicating their commercial viability [1,2,3,4,5]. The inclusion of poisonous lead (Pb) poses significant environmental issues for the prospective commercialization of perovskite solar cells [6,7,8], despite the amazing advances made. Serious and collaborative efforts are being made to discover, evaluate, design, and synthesize lead-free perovskite materials with superior photovoltaic performance. Numerous lead-free perovskite compounds containing non-toxic elements such as Ge (germanium), Sn (tin), Sb (antimony), Cu (copper), Bi (bismuth), etc., have already been reported [9,10,11,12]. However, the majority of these lead-free metal-based halide perovskites have not performed well to date, and their stated power-conversion efficiency is much below that of the champion methylammonium lead trihalide (CH_3_NH_3_PbX_3_, where X = I, Br or Cl) [13,14,15,16,17]. Among these lead-free metals for perovskites, the Sn-based perovskite solar cell has performed excellently, and their maximum reported efficiency has already reached a little above 10%. Both Sn and Pb share many similarities such as electronic structure, binding energy, and ionic radius which can facilitate Sn to replace Pb for perovskite solar cells [18,19,20,21]. Therefore, comprehensive investigations are required to further improve their power-conversion efficacy to replace champion methylammonium lead trihalide for photovoltaic applications.

As noted previously, the power-conversion efficiency of Sn-based perovskite solar cells is still inferior to that of the extremely efficient Pb-based perovskite solar cell. Numerous hypotheses have been presented to explain the comparatively poor performance of Sn-based perovskite solar cells, especially with device degradation attributed to the transformation of unstable Sn^+2^ ions into stable Sn^+4^ ions under ambient environmental conditions. As a result of the low formation energy of the stable states, the transition of Sn^+2^/Sn^+4^ generates some vacancies. These vacancies behave as p-type self-doping and create the source of recombination centers that reduce the photovoltaic response of Sn-based perovskite solar cells, very similar to polymer solar cells [21,22].

ASnI_3_ types of perovskites have been regarded as the best alternative for lead-free photovoltaic devices among the various Sn-based perovskites [23,24,25], where A is a cation selected from MA (CH_3_NH_3_, methylammonium), FA (CH(NH_2_)_2_, formamidinium), MP (CH_3_PH_3_, methyl phosphonium), Cs (cesium metal), and/or their mixtures. Recent reports have identified mixed-cation tin halide as a highly promising perovskite for efficient solar devices. In SnI_3_, mixed cations, such as formamide-cesium cations (FACs)+, serve a dual role. On the one hand, mixed cations (especially 85% of FA and 15% of Cs) stabilize the perovskite, and on the other hand, they prevent vacancy formation, reduce the trap density for electron-hole recombination, improve the hysteresis, provide very high shunt-resistance, enhance charge collection, and thus improve the overall photovoltaic response of the given perovskite solar cell [25,26,27,28]. Liao and his colleagues have achieved FASnI_3_-based perovskite solar cells with close to 6% power-conversion efficiency [29], where PEDOT:PSS was used as the hole-transport layer, and C60 was employed as the electron-transport layer. Shao et al. [27] produced a planar perovskite solar cell based on FASnI_3_ with a maximum power-conversion efficiency of up to 9.0%. Similarly, Li and his colleague recently reported that the FASnI_3_-based device with PEDOT:PSS as the hole-transport layer and C60/BCP as the electron-transport layer had the highest power-conversion efficiency of 10.4% [30]. Table 1 displays the performance of the FASnI_3_-based perovskite solar cell over all important milestones.

Despite these successes, FACsSnI_3_ is still a relatively novel perovskite with insufficient knowledge of the hole and electron-transport layers’ requirements for efficient photovoltaic performances. The primary objective of this study is to investigate FACsSnI_3_-based perovskite solar cells using simulations and modeling to optimize the material parameters, such as electron affinity, energy bandgap, film thickness, and doping density of both the hole and electron-transport layers, that provide the highest power-conversion efficiency.

## 2. Device Modeling and Simulation Methods

### 2.1. Simulation Device Models

The device simulation software for solar cells typically solves a sequence of coupled device differential equations using standard mathematical approaches. These solutions will determine the general-purpose photovoltaic responses of any type of solar cell, including open-circuit voltage, short-circuit current, fill factor, and power-conversion efficiency. These models of photovoltaic devices can be classified as [37,38,39,40]:

#### 2.1.1. Poisson Model

The Poisson model describes the underlying behavior of electronic potential (ϕ), which depends on all key charge parameters and their allocation inside the solar thin film. In the Poisson model, the electronic charge is denoted by “*e*”, with a typical value of 1.602 × 10^−19^ C, and both absolute dielectric constant and relative dielectric constant are denoted by ”$\epsilon_{o}$“ and “$\epsilon_{r}$”, respectively, for each corresponding thin-film material. Similarly, the shallow donor density and shallow acceptor density are represented by “*N_D_*” and “*N_A_*”, respectively. While “ρ_p_ “ and “ρ_n_ “ are used to define the free density distribution of hole and electron, respectively, and the position (*x*) dependence hole and electron distributions are denoted by “*p*(*x*)” by “*n*(*x*)”, respectively. Therefore, the Poisson model can be described as follows:

(1) $$\frac{d^{2}\phi \left(x\right)}{dx^{2}}=\frac{e}{\epsilon_{o}\epsilon_{r}}\;\left(p\left(x\right)-n\left(x\right)+N_{D}-N_{A}+\rho_{p}-\rho_{n}\right)$$

#### 2.1.2. Continuity Model

Another important photovoltaic device model is the continuity model. The continuity model provides the relation between the first-order derivative of electron and hole current density “*J_n_*(*x*)” and “*J_p_*(*x*)” (function of position (*x*)) and free electron-hole generation (*G*) as well as recombination (*R*). The continuity model can be expressed as follows:

(2) $$\frac{dJ_{n}}{dx}=G-R$$

(3) $$\frac{dJ_{p}}{dx}=G-R$$

#### 2.1.3. Charge Transport Model

Any photovoltaic device is a semiconductor *p-i-n* junction diode in nature. As a result, all charge transport models which apply to the *p-i-n* junction diode can also be used for photovoltaic solar cells and are available in the simulation software. Here, one of the fundamental charge transport models is selected and listed below. In this charge transport model, it is defined that overall current is the resultant of both electron “*J_n_*” and hole “*J_p_*” current density and each current density is also the result of drift and diffusion current of holes as well as electrons. For drift and diffusion current, “*µ_p_* “ and “*µ_n_* “ are defined as the mobility of the hole and electron, respectively, and similarly for the diffusion current, “*D_p_*” and “*D_n_*” are the hole and electron diffusion coefficient. Finally, the charge transport model for the solar cell can be written as follows:

(4) $$J=J_{n}+J_{p}$$

(5) $$\;J_{n}=D_{n}\;\frac{dn}{dx}+\mu_{n\;}n\frac{d\phi }{dx}$$

(6) $$J_{p}=-D_{p}\;\frac{dp}{dx}+\mu_{p\;}p\frac{d\phi }{dx}$$

#### 2.1.4. Optical Absorption Model

For the optical absorption model, we select the traditional model from a list of optical absorption models available in SCAPS-1D. In the traditional model, the optical absorption coefficient “α” is a function of optical wavelength “*λ*” with energy “*hυ*” and can be defined as *α(λ)*. In this model, as defined by Equation (7), both *A* and *B* are considered arbitrary constants, and “*E_g_*” is the energy bandgap of the corresponding thin-film layer. The traditional optical model can be written as

(7) $$\alpha \;(\lambda )=\left(A+\frac{B}{h\nu }\right)\;\sqrt{h\nu -E_{g}}$$

### 2.2. Simulation Software

The simulation and modeling process is a science as well as an art, and it offers numerous advantages. Now it is the foundation of the contemporary photovoltaic industries. Since the invention of the first semiconductor solar cell, simulation, and modeling software for solar cells have been practiced and documented successfully with constant improvement. Modern simulation software can predict the experimental findings of a solar cell with outstanding precision [41,42]. However, it depends on a number of factors, including the following:

(i) The nature of the simulation software.
(ii) The capacity of the simulation software.
(iii) The variety of available models.
(iv) The well-defined workflow to execute the task.
(v) The numerical approaches used to solve the models.
(vi) The defined accuracy of the internal and external variables.
(vii) The user-friendliness of the software.
(viii) The continuous improvement feedback cycle.

SCAPS 1D, an open-source simulation program, satisfies several of these criteria. Therefore, SCAPS-1D was carefully selected for this investigation since it is well-documented and supported by numerous industrial and academic experts on perovskite solar cells. This simulation uses SCAPS-1D version 3.3.10, which was produced by a team led by Marc Bargeman at the University of Gent, Belgium [38,39,40,41,42,43,44,45].

### 2.3. Design of Proposed Solar Cell

Perovskite-type solar cells can be broadly classified into two well-defined designs. These architectures for solar cells are either *n-i-p* (conventional or non-inverted) or *p-i-n* (inverted or non-conventional). The primary distinction between these two architectures is the nature of the transport layer (either an electron layer for *n-i-p* architecture or a hole layer for *p-i-n* architecture) directly facing the solar spectrum on top of the transparent electrode. Generally, n-i-p-type solar cells demonstrate the best power-conversion efficiency [46,47]. However, numerous researchers have lately discovered that *p-i-n* architecture has various benefits, such as enabling device production at low temperatures, boosting operational stability, and facilitating integration with other conventional and new solar-cell technologies. Therefore, *p-i-n* architecture is selected for the study of the proposed FACsSnI_3_-based perovskite solar cell [48,49].

### 2.4. Simulation Materials Parameters

The photovoltaic response obtained from general-purpose device models is dependent on the correctness of the material properties of each constituent layer. The absorber FACsSnI_3_ is a novel perovskite about which there is limited and varied information available in the literature. After a comprehensive examination of the relevant literature, the material properties of the absorber layer and the probable range of reported thickness are extracted and listed in Table 2 with the corresponding references.

The absorber FACsSnI_3_, like many other perovskites, is also suffering from switchable photo-current, hysteresis, and sluggish photovoltaic response, and the dynamics of defect migration are the possible reasons for such unavoidable responses [50,51]. Where defects are produced inside perovskite due to imperfections during the fabrication process, disorders of molecular structure, interface dangling bonds, grain boundaries, and many other reasons [52,53,54]. Therefore, a defects density, which is distributed uniformly with a 10^14^ cm^2^ electron and hole capture cross-section, is introduced into the bulk region of perovskite as shown in Table 2. The main objective of this study is to determine the ideal hole and electron-transport layer’s parameters, which give the maximum photovoltaic response. Therefore, random parameters within the possible ranges are introduced in the simulation for both the hole and electron-transport layer. Detailed information can be found in [54,55].

**Table 2:** The random simulation parameters are used for the hole-transport layer and electron-transport layer, while simulation parameters for active perovskite layers FACsSnI_3_ (FA_0.85_Cs_0.15_SnI_3_) were taken from the given references.

Photovoltaic Parameters	Symbol	Unit	Hole-Transport Layer	Electron-Transport Layer	FACsSnI_3_
Thickness	Th	nm	200	200	200
Energy Band Gap	*E_g_*	eV	2	3.4	1.45
Electron Affinity	*χ*	eV	3	4	4.1
Dielectric Permittivity	*ϵ_r_*		18	9	9
Effective Density of States at Conduction Band	*N_c_*	cm^−3^	2 × 10^20^	2 × 10^20^	6 × 10^18^
Effective Density of States at Valence Band	*N_v_*	cm^−3^	2 × 10^20^	2 × 10^20^	2.14 × 10^19^
Hole Thermal Velocity	*V_h_*	cm/s	1 × 10^7^	1 × 10^7^	1 × 10^7^
Electron Thermal Velocity	*V_e_*	cm/s	1 × 10^7^	1 × 10^7^	1 × 10^7^
Electron Mobility	*µ_e_*	cm^−2^/V·s	4 × 10^−4^	200	2.36 × 10^−1^
Hole Mobility	*µ_h_*	cm^−2^/V·s	4 × 10^−4^	80	1.7 × 10^−1^
Uniform Shallow Donor Doping	*N_d_*	cm^−3^	-	1 x10^16^	1 × 10^19^
Uniform Shallow Acceptor Doping	*N_a_*	cm^−3^	1 x10^16^	-	1 × 10^19^
Defect Density	*N_t_*	cm^−3^	1 × 10^14^	1 × 10^14^	1 × 10^14^
References					[56,57,58,59,60]

### 2.5. Simulation Flowchart

**Figure 1:**
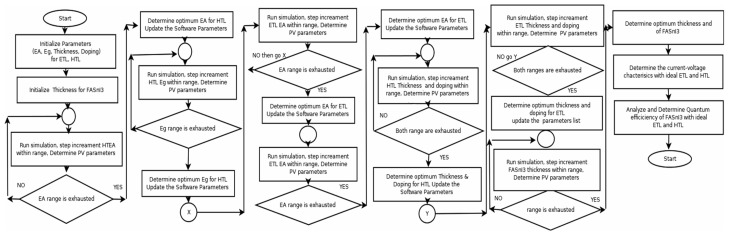
Shows the flowchart to determine the ideal electron and hole-transport parameters for FACsSnI_3_-based perovskite solar cell.

The steps needed to establish the optimal parameters for the electron and hole-transport layers of a FACsSnI_3_-based perovskite solar cell are outlined below and depicted in Figure 1.

- Step 1: Determine the range of some parameters, such as electron affinity (EA), energy bandgap (*Eg*), film thickness, and doping density, for the hole-transport layer (HTL), and the electron-transport layer (ETL), using the literature [55,57] as a reference.
- Step 2: Estimate the range of thickness for the FACsSnI_3_ absorber layer from the literature [56,61].
- Step 3: Take random values from the range of steps 1 and 2 for the parameters listed above. Other parameters should be set to standard values with given references [53,54,55,56,57,58,59,60,61,62].
- Step 4: Set the thickness of the FACsSnI_3_ absorber layer to a minimum and gradually increase it up to the maximum of the range.
  o Step 4a: Determine the optimum EA of the hole-transport layer through simulation, which gives max PCE. Update the simulation parameter for electron affinity (hole-transport layer).
  o Step 4b: Determine the optimum *Eg* of the hole-transport layer, which gives max PCE. Update the simulation parameter for *Eg* (hole-transport layer).
  o Step 4c: Determine the optimum EA of the electron-transport layer, which gives max PCE. Update the simulation parameter for EA (electron-transport layer).
  o Step 4d: Determine the optimum *Eg* of the electron-transport layer, which gives max PCE. Update the simulation parameter for *Eg* (electron-transport layer).
  o Step 4e: Determine the optimum thickness of the hole-transport layer, which gives max PCE. Update the simulation parameter for thickness (hole-transport layer).
  o Step 4f: Determine the optimum *Na* of the hole-transport layer, which gives max PCE. Update the simulation parameter for *Na* (hole-transport layer).
  o Step 4g: Determine the optimum thickness of the electron-transport layer, which gives max PCE. Update the simulation parameter for thickness (electron-transport layer).
  o Step 4h: Determine the optimum *Nd* of the electron-transport layer, which gives max PCE. Update the simulation parameter for *Nd* (electron-transport layer).
  o If the thickness loop of the FACsSnI_3_ is not exhausted, then go to Step 4a.
- Step 5: Determine the thickness of FACsSnI_3_ which gives the maximum PCE with ideal hole-transport layer and electron-transport layer parameters.
- Step 6: Determine the photocurrent-voltage response of the optimized device with an ideal hole-transport layer and electron-transport layer.
- Step 7: Determine the other responses of the optimized device with an ideal hole-transport layer and electron-transport layer.
- End of simulation.

## 3. Results and Discussion

### 3.1. Optimization of Electron Affinity for Hole-Transport Layer

After the initialization of the simulation parameter, the first step is to determine the most suitable value of the electron affinity (Χ) of the ideal hole-transport layer for the FACsSnI_3_-based perovskite solar cell. The electron affinity of the hole-transport layer plays a vital role in extracting holes and at the same time blocks the electron from the active FACsSnI_3_ perovskite layer. The electron affinity is the energy difference between the vacuum energy level and the LUMO (lowest unoccupied molecular or conduction band) energy level of the hole-transport layer. From the literature, it is observed that the possible value of electron affinity should be between 1.5 eV and 4.0 eV for most of the hole-transport materials and especially for the perovskite absorber layer.

**Figure 2:**
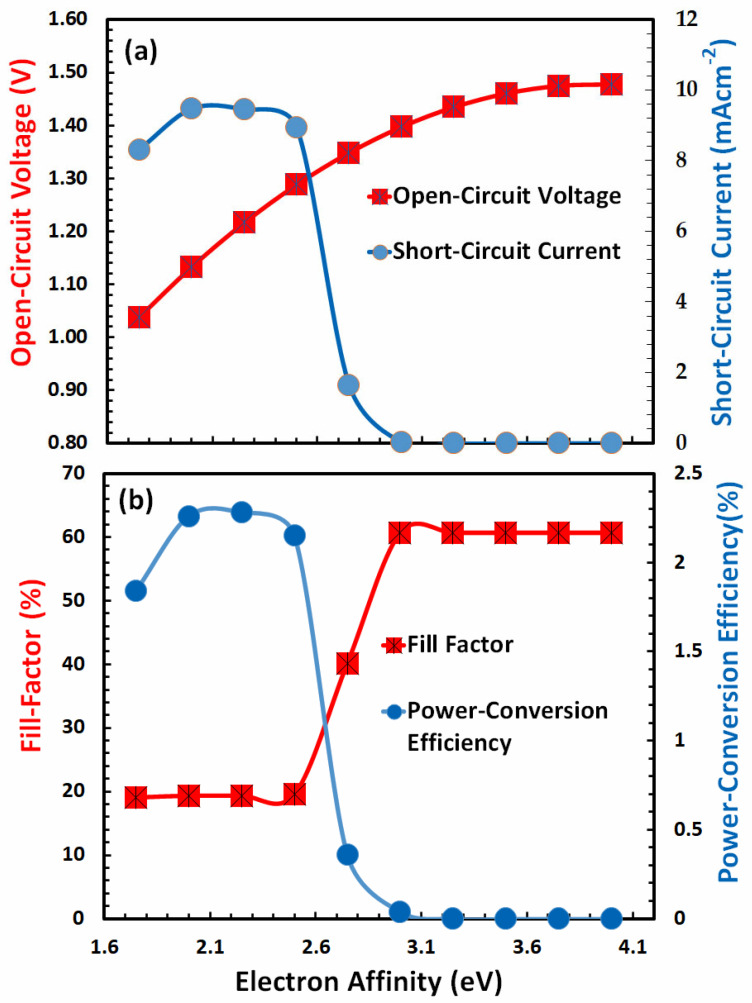
Shows the photovoltaic parameters (**a**) open-circuit voltage, short-circuit current, (**b**) fill factor, and power-conversion efficiency of FACsSnI_3_-based perovskite solar cell as a function of the electron affinity (Χ) of an ideal hole-transport layer.

Figure 2a,b show the photovoltaic parameters of the FACsSnI_3_-based perovskite solar cell as a function of the electron affinity (Χ) of the ideal hole-transport layer, which is determined from the series of simulations. Figure 2a reveals that the open-circuit voltage linearly increases and then becomes nearly constant at higher electron affinity of the hole-transport layer, whereas the short-circuit current of the solar increases up to 2.2 eV of electron affinity and then begins to decrease sharply and reaches its minimum after 3 eV or more of the electron affinity of the hole-transport layer. Similar to Figure 2a,b demonstrates that the fill factor begins very slowly, makes a sharp rise at around 2.6 eV, and then remains practically constant. As noted previously, the behavior of power-conversion efficiency as a function of electron affinity is very similar to that of short-circuit current, with a maximum of 2.12 eV of the electron affinity of the hole-transport layer. Therefore, it can be estimated that 2.12 eV is the most optimal electron affinity for the hole-transport layer and then updated in the simulation software for further simulations.

### 3.2. Optimization of Energy Bandgap for Hole-Transport Layer

**Figure 3:**
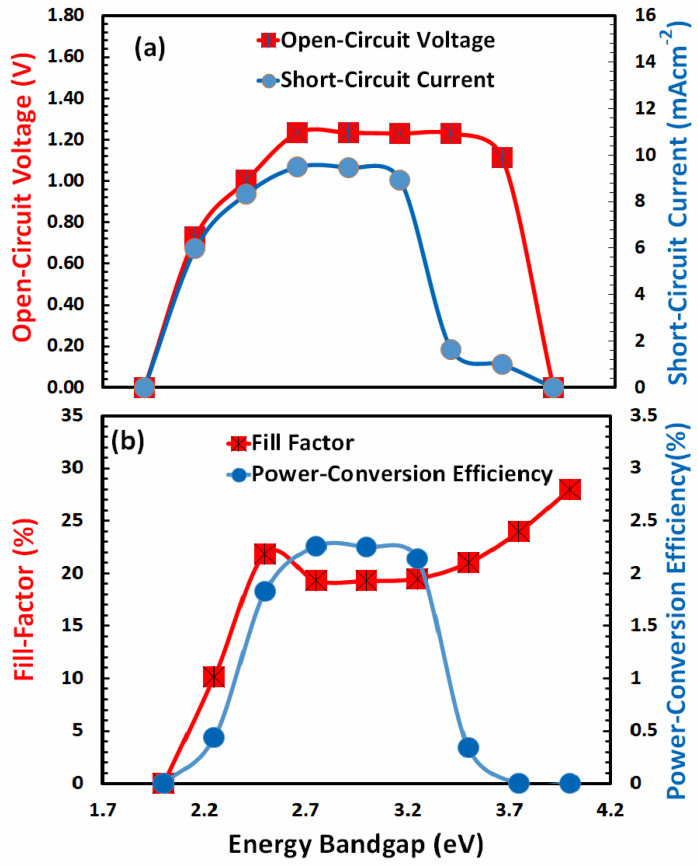
Shows the photovoltaic parameters (**a**) open-circuit voltage, short-circuit current, (**b**) fill factor, and power-conversion efficiency of FACsSnI_3_-based perovskite solar cell as a function of the energy bandgap (*E_g_*) of an ideal hole-transport layer.

Another important parameter that influences the overall photovoltaic response is the energy bandgap of the hole-transport layer. For efficient photovoltaic response, the energy band gap of the hole-transport layer (i) should be transparent to most of the falling photons, (ii) should offer a low energy barrier to extract holes from the absorber, (iii) should have the ability to block the injection of electrons, and (iv) should present low potential barrier with anode for efficient charge collection. Thus, for the optimal photovoltaic response of FACsSnI_3_-based solar cells with an excellent hole-transport layer, the energy band gap needs to be optimized. Figure 3 shows the behavior of photovoltaic parameters, such as open-circuit voltage and short-circuit current in Figure 3a, while fill factor and power-conversion efficiency in Figure 3b as a function of the energy bandgap of an ideal hole-transport layer.

The figures reveal that, with the exception of the fill factor, all photovoltaic parameters exhibit a comparable response as a function of the energy band gap. At an energy bandgap of about 2 eV, all of these photovoltaic parameters are close to zero. These values grew, peaked between 2.7 and 3 eV, and subsequently fell quickly at a higher energy bandgap. The fill-factor behavior is quite complex with respect to the energy band gap of the hole-transport layer. Due to the fact that power-conversion efficiency is the most crucial factor, it is most significant at 2.7 eV. Therefore, it can be concluded that the overall hole-transport layer is optimized at an electron affinity of 2.12 eV and an energy bandgap of 2.7 eV of the hole-transport layer.

### 3.3. Optimization of Electron Affinity for Electron-Transport Layer

**Figure 4:**
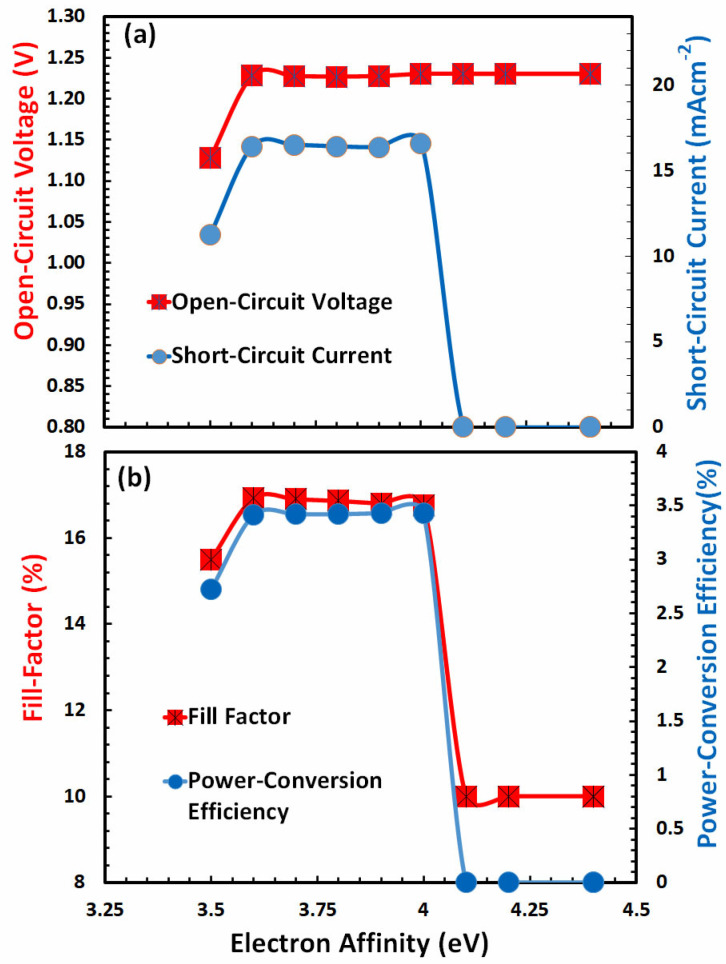
Shows the photovoltaic parameters (**a**) open-circuit voltage, short-circuit current, (**b**) fill factor, and power-conversion efficiency of FACsSnI_3_-based perovskite solar cell as a function of the electron affinity (Χ) of an ideal electron-transport layer.

The electron affinity of the electron-transport layer is just as crucial as that of the hole-transport layer, as mentioned previously. In general, the electron affinity of the electron-transport layer should be larger than that of FACsSnI_3_ (−4 eV) for the successful extraction of electrons from the perovskite layer. After incorporating the optimal values of electrical affinity and energy bandgap of the hole-transport layer into the simulations, the electron affinity of the electron-transport layer is computed, and the results are displayed in Figure 4a and Figure 4b, respectively.

Figure 4a,b illustrate that, with the exception of open-circuit voltage, which remains constant after 3.6 eV, all other photovoltaic parameters behave identically with respect to the ionization potential of the electron-transport layer. All of these characteristics escalate somewhat at first, remain almost constant up to 4 eV of *E_g_*, and then decline dramatically beyond 4 eV of the electron-transport layer’s electron affinity. As power-conversion efficiency hits its maximum at 3.6 eV, the optimal value of the electron affinity of the electron-transport layer can be estimated at 3.6 eV.

### 3.4. Optimization of Energy Bandgap for Electron-Transport Layer

**Figure 5:**
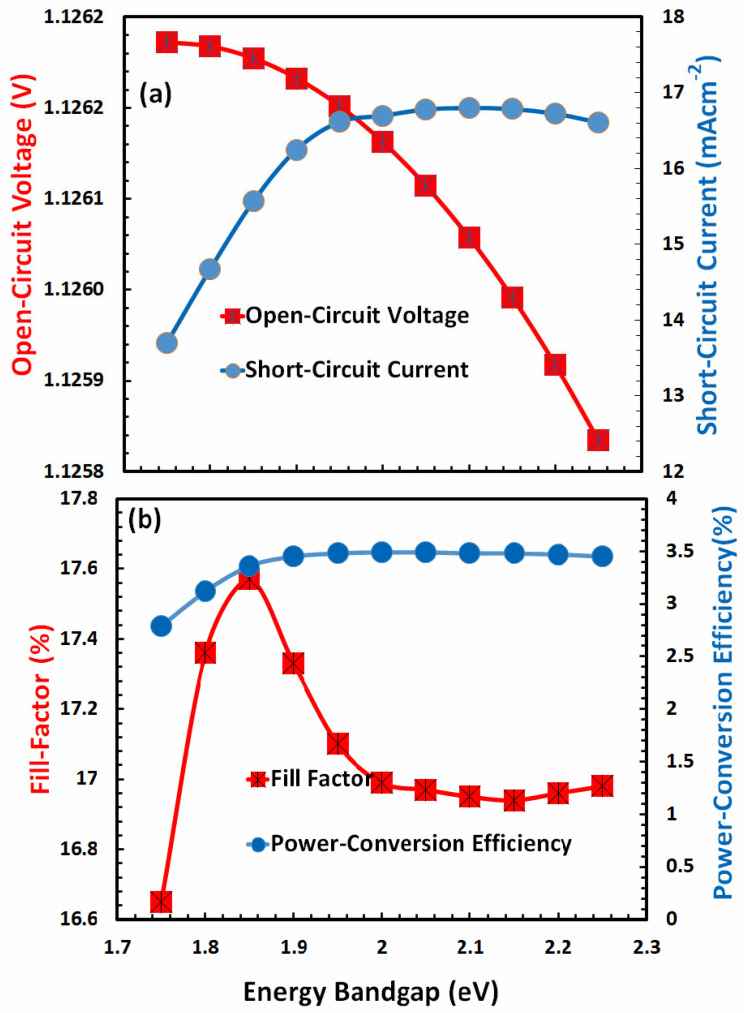
Shows the photovoltaic parameters (**a**) open-circuit voltage, short-circuit current, (**b**) fill factor, and power-conversion efficiency of FACsSnI_3_-based perovskite solar cell as a function of the energy bandgap (*E_g_*) of an ideal electron-transport layer.

The energy band gap becomes significant after adjusting electron affinity in order to optimize several complex parameters for an efficient photovoltaic response. Some of these parameters include preventing perovskite hole injection into the electron-transport layer, avoiding optical absorption, assisting maximum optical reflection toward the perovskite absorption layer, reducing recombination losses at interfaces, and promoting efficient electron transport [63]. Among these characteristics, blocking of opposing carriers is critical, and it can be improved by lowering the HOMO (Highest Occupied Molecular Orbital or valence band) of the electron-transport layer in comparison to the HOMO (5.45 eV) of the perovskite absorber layer. This, in turn, facilitates the prevention of holes from the absorber layer being injected into the electron-transport layer; otherwise, the injected holes can recombine with electrons in the electron-transport layer, degrading the total photovoltaic response. The photovoltaic parameters are derived as a function of the energy band gap in order to maximize the energy band gap for the HOMO level of the electron-transport layer, as illustrated in Figure 5a,b.

According to Figure 5a, when the energy bandgap of the electron-transport layer increases, open-circuit voltage continuously decreases (within a very short range) and reaches its lowest point at 2.25 eV. This could be due to the growing conduction band offset between the electron-transport layer and the FACsSnI_3_ layer. Both the short-circuit current (see Figure 5a) and the fill factor (see Figure 5b) start to increase first and then decrease when the electron affinity reaches a certain level, while the short-circuit current and power-conversion efficiency do so very gradually for the former and quite rapidly for the latter. Since it provides the highest level of power-conversion efficiency, 2 eV is the most optimal energy bandgap for the electron-transport layer. The optimized value is then updated in the software for subsequent simulations.

### 3.5. Optimization of Film Thickness for Hole-Transport Layer

**Figure 6:**
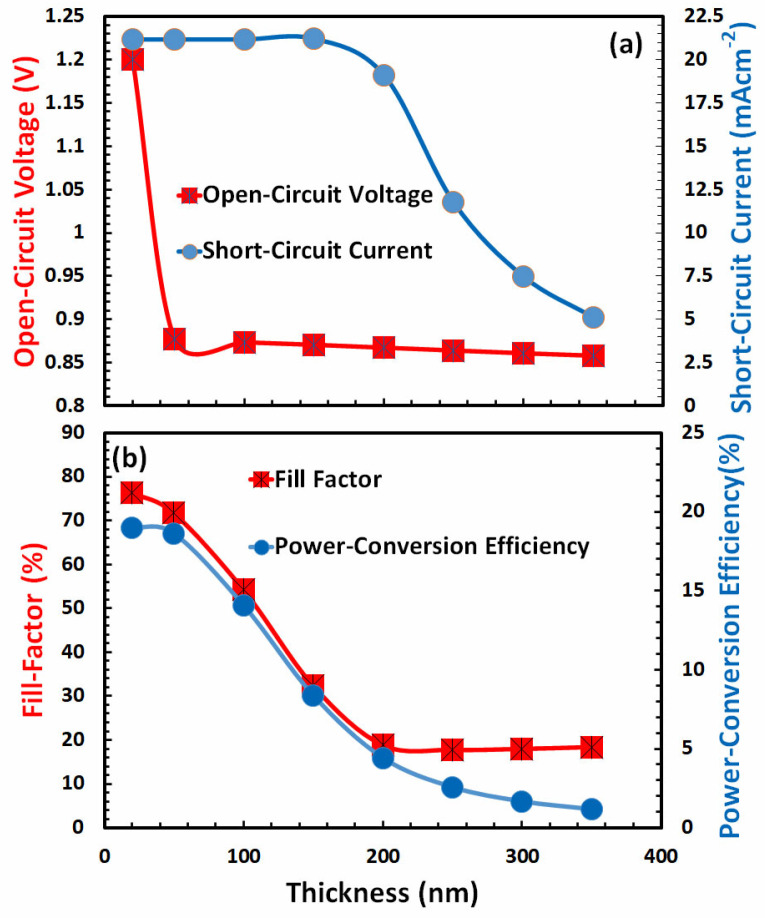
Shows the photovoltaic parameters (**a**) open-circuit voltage, short-circuit current, (**b**) fill factor, and power-conversion efficiency of FACsSnI_3_-based perovskite solar cell as a function of the film thickness of an ideal hole-transport layer.

The optimization of a physical parameter, like the thickness of a hole-transport layer, is another important task. The optical transparency of the absorber, charge extraction from the absorber, hole transport in the bulk region, hole collection by anodes from the hole-transport layer, and many other factors at once are all factors that affect the thickness optimization of the hole-transport layer for the *p-i-n* type perovskite like many other types of polymer solar cells [64,65,66,67]. In order to determine the ideal thickness of the hole-transport layer, the photovoltaic parameters are calculated as a function of film thickness and results are illustrated in Figure 6a,b.

All the photovoltaic characteristics have a close connection to the thickness of the hole-transport layer, as shown in Figure 6a,b. When the thickness of the hole-transport layer is increased, there is a corresponding decline in all of these photovoltaic parameters. It is well reported that both optical absorption and hole transport in the bulk region deteriorated as a result of the increased thickness of the hole-transport layer [68]. So, it can be seen in Figure 6a,b, the power-conversion efficiency is at its highest when the thickness of the hole-transport layer is 20 nanometers. Therefore, it is possible to draw the conclusion that a thickness of 20 nm for the hole-transport layer is the optimal thickness for the suggested FACsSnI_3_-based perovskite solar cell.

### 3.6. Optimization of Doping Density for Hole-Transport Layer

**Figure 7:**
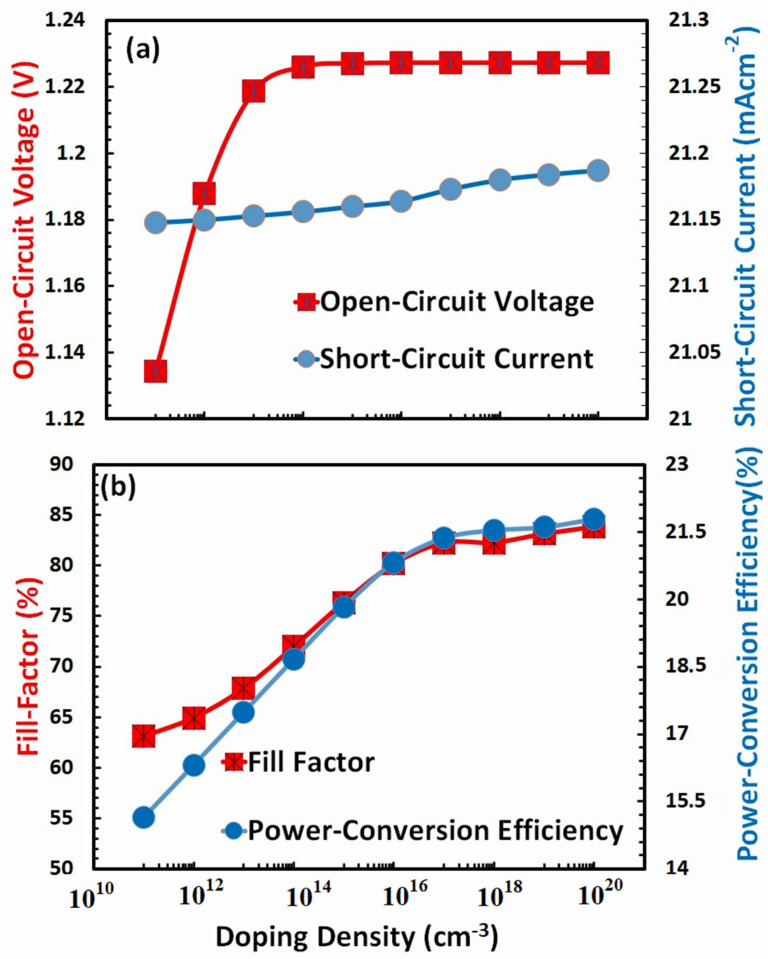
Shows the photovoltaic parameters (**a**) open-circuit voltage, short-circuit current, (**b**) fill factor, and power-conversion efficiency of FACsSnI_3_-based perovskite solar cell as a function of the doping density of an ideal hole-transport layer.

It is generally accepted that the photovoltaic characteristics, in many respects, directly depend on the doping of both the hole-transport layer and the electron-transport layer. Heavy doping increases the hole-transport layer’s conductivity, improving the overall charge transport process. It also provides ohmic contact with the electrode, which is necessary for the efficient collection of charges, and it improves the interface resistance by reducing traps in the perovskite layer, which is necessary for the efficient extraction of holes. Tuning the energy level between the hole-transport layer and the absorber, as well as the energy level between the anode and the hole-transport layer, can be facilitated by the optimized doping of the hole-transport layer. On the other hand, excessive amounts of dopant atoms can result in phenomena such as parasitic absorption, Moss-Burstein effects, a reduction in photovoltaic response, and even doping atoms may become the sources of recombination [69,70]. Therefore, to achieve an efficient photovoltaic design for a FACsSnI_3_-based perovskite solar cell, it is required to optimize the doping density of the hole-transport layer. The photovoltaic response of a perovskite solar cell based on FACsSnI_3_ is shown in Figure 7 as a function of the doping density in the hole-transport layer.

All photovoltaic parameters, such as Figure 7a open-circuit voltage and short-circuit current, as well as Figure 7b fill factor and power-conversion efficiency, are seen to increase with higher doping density, as was to be predicted, as shown by both figures. It has been determined that a doping density of 10^20^ cm^3^ in the hole-transport layer is the optimal density for the FACsSnI_3_-based perovskite solar cells.

### 3.7. Optimization of Film Thickness for Electron-Transport Layer

**Figure 8:**
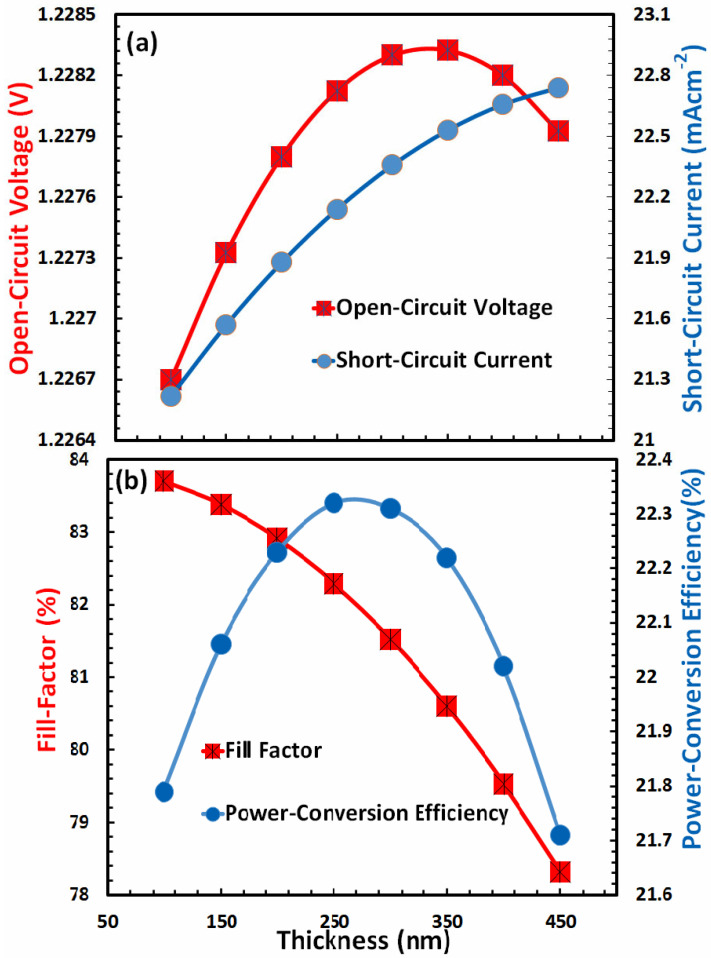
Shows the photovoltaic parameters (**a**) open-circuit voltage, short-circuit current, (**b**) fill factor, and power-conversion efficiency of FACsSnI_3_ -based perovskite solar cell as a function of the film thickness of an ideal electron-transport layer.

In the majority of cases, it has been observed that when the electron-transport layer thickness of perovskite solar cells increases, the photovoltaic parameters’ efficiency also increases, reaches a maximum at a specific optimum thickness, and subsequently declines [67]. It may be due to a number of factors requiring optimization, including (i) electron transport, (ii) electron collection at the cathode, (iii) electron extraction from the absorber, (iv) blocking of the hole from the absorber, (v) series resistance of the bulk, (vi) electron-hole recombination in the bulk, (vii) optical transmission and absorption, and numerous other factors [70,71]. As depicted in Figure 8a,b, similar responses are observed when determining open-circuit voltage, short-circuit current, fill factor, and power-conversion efficiency as a function of electron-transport layer thickness.

Figure 8a for a FACsSnI_3_-based perovskite solar cell demonstrates that the open-circuit voltage increases achieve a maximum, and then begins to fall, but the short-circuit current increases and may reach a maximum beyond the 450 nm thickness of the electron-transport layer. Figure 8b demonstrates that the fill factor reaches its maximum before 75 nm thickness and therefore decreases with thickness for the given range, whereas the power-conversion efficiency reaches its maximum at 260 nm thickness of the electron-transport layer for a FACsSnI_3_-based perovskite solar cell. Therefore, 260 nm is the optimal electron-transport layer thickness for a FACsSnI_3_-based perovskite solar cell.

### 3.8. Optimization of Doping Density for Electron-Transport Layer

**Figure 9:**
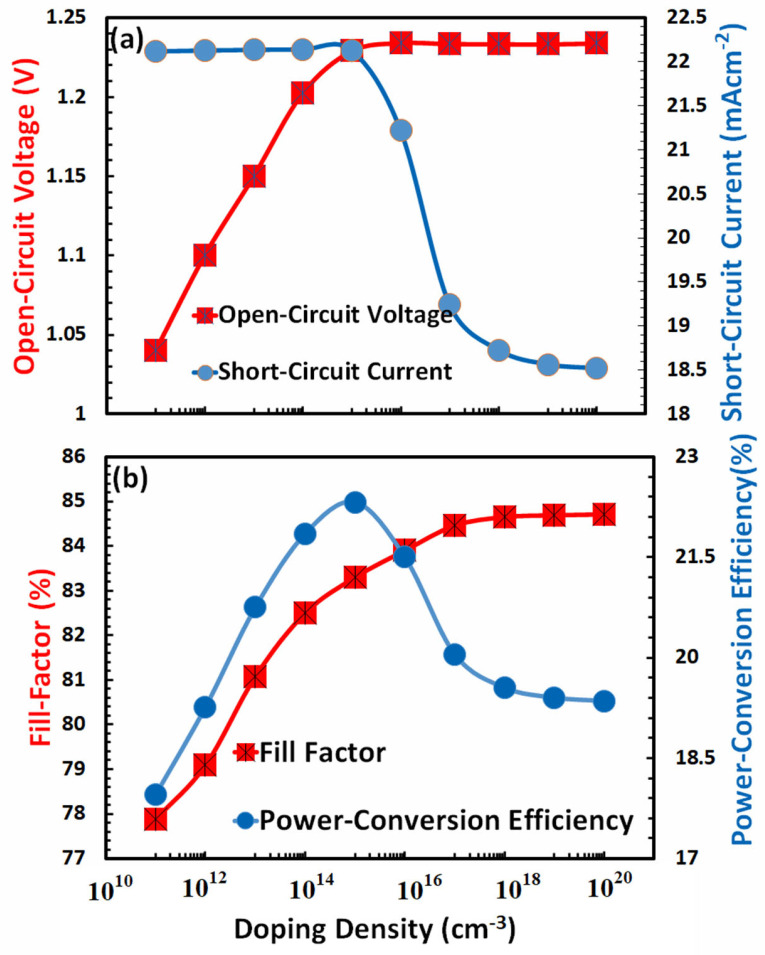
Shows the photovoltaic parameters (**a**) open-circuit voltage, short-circuit current, (**b**) fill factor, and power-conversion efficiency of FACsSnI_3_-based perovskite solar cell as a function of the doping density of an ideal electron-transport layer.

As with the hole-transport layer, the proper doping of the electron-transport layer is also very crucial for enhancing the electron charge transport. Similarly, it is correspondingly important to improve the quality of the interface between the electron-transport layer and perovskite absorption layer as well as with the cathode layer. The high-quality interface helps to achieve efficient charge transport in the bulk region and minimize traps at their interfaces with perovskite and the cathode layer. Similarly, excessive doping also results in parasitic absorption, hysteresis, stability problems, Moss-Burstein effects, and numerous other unwanted issues [72]. Figure 9 demonstrates the behavior of photovoltaic parameters such as (a) open-circuit voltage, short-circuit current, and (b) fill factor, power-conversion efficiency as a function of doping density of the electron-transport layer for a FACsSnI_3_-based perovskite solar cell. Open-circuit voltage and short-circuit current behave differently, as short-circuit current increases with the increase in doping density, while open-circuit voltage decreases with the increase in doping density. On the other hand, power-conversion efficiency and fill factor behave very similarly before 10^16^ cm^3^ doping density, and then power-conversion efficiency starts to decrease. The optimal doping density of the electron-transport layer for a FACsSnI_3_-based perovskite solar cell is 10^16^ cm^3^ given that the power-conversion efficiency is maximum at this doping density.

### 3.9. Optimization of Thickness for FACsSnI_3_ Absorber Layer

Like many other organic or polymer semiconductors, p-type self-doping is a severe problem that FACsSnI_3_-type perovskites encounter [73,74,75]. Such self-doping effects result in shallow trap formation, shortening carrier diffusion lengths, and reducing carrier lifetimes. The density of these traps sharply increases as the thickness of the absorber layer increases. On the other hand, optical absorption is inadequate, necessitating a thick perovskite layer for sufficient optical absorption [21,76]. Therefore, the thickness optimization of the FACsSnI_3_ becomes a challenging task for the efficient photovoltaic response.

**Figure 10:**
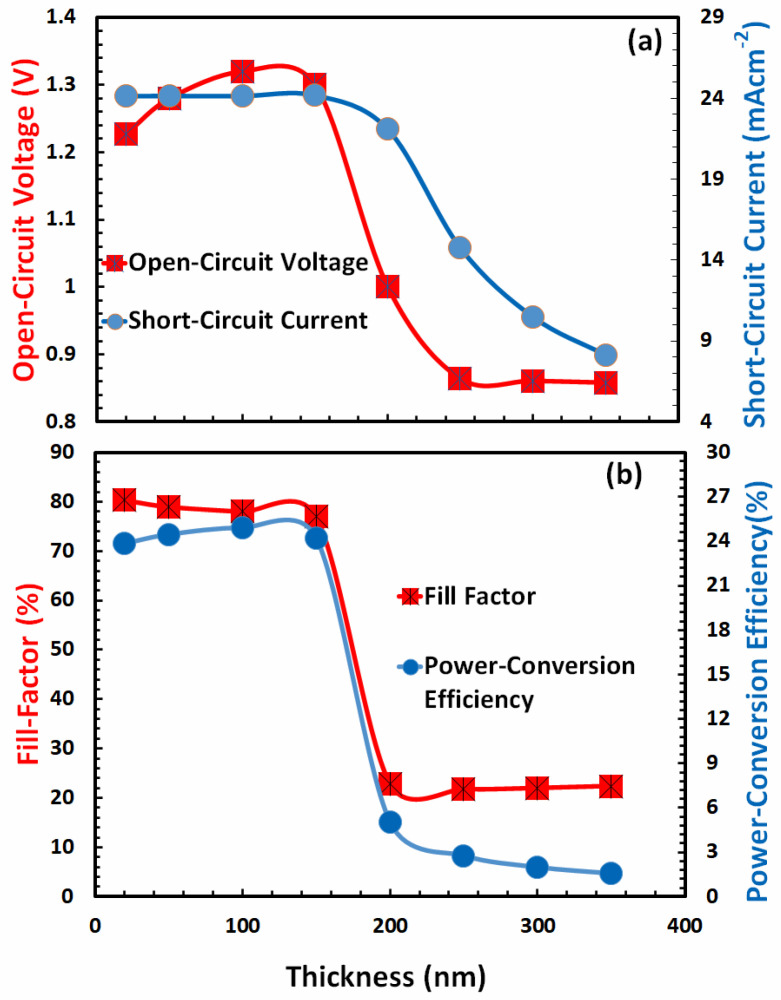
Shows the photovoltaic parameters (**a**) open-circuit voltage, short-circuit current, (**b**) fill factor, and power-conversion efficiency of FACsSnI_3_-based perovskite solar cell as a function of the film thickness of FACsSnI_3_ absorber layer.

In order to identify the optimal thickness of the absorber perovskite layer, the photovoltaic characteristics of the proposed devices are determined as a function of FACsSnI_3_ layer thickness, as shown in Figure 10a,b. All photovoltaic parameters exhibit highly similar responses as a function of the thickness of the absorber layer film. In Figure 10a, the open-circuit voltage decreases after reaching a certain maximum due to the high trap density at a higher thickness of the absorber layer. As the absorber layer’s thickness increases, the short-circuit current reaches its maximum value and then begins to fall. Figure 10b illustrates that fill factor and power-conversion efficiency exhibit responses that are comparable to one another. Because the power-conversion efficiency is a decisive parameter, and because it reaches its highest value at 100 nm, it is possible to deduce that 100 nm is the optimal thickness of absorber FACsSnI_3_ for the efficient response of the proposed perovskite solar cell.

### 3.10. Overall Photovoltaic Response of the Optimized Solar Cell

**Figure 11:**
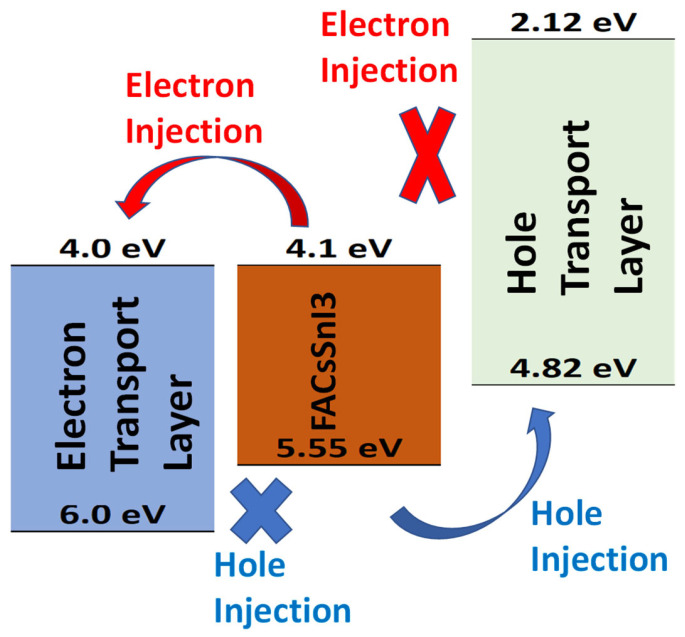
Band alignment architecture of the optimized device where FACsSnI_3_ as perovskite is sandwiched between electron and hole-transport layers.

**Figure 12:**
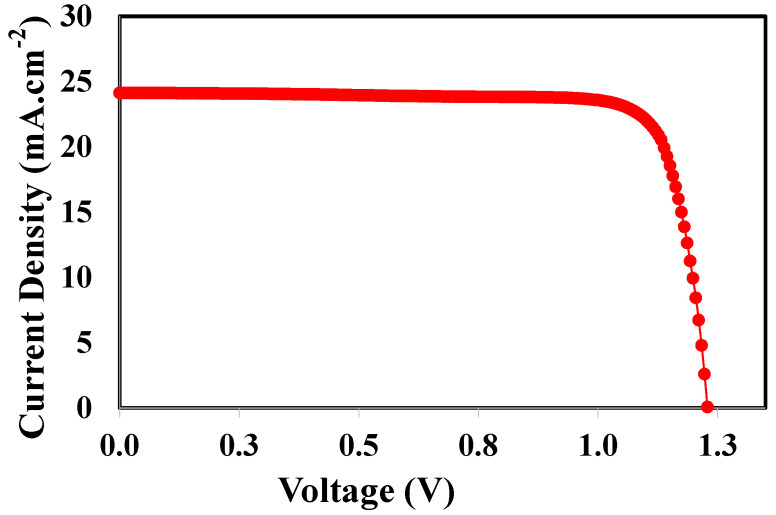
The overall photovoltaic response of the optimized FACsSnI_3_-based perovskite solar cell.

For the FACsSnI_3_-based perovskite solar cell, Figure 11 depicts the optimized band alignment of the hole and electron-transport layer [77]. Table 3 lists all of the optimized parameters, including the layer’s thickness, electron affinities, energy band gaps, and doping densities that were determined through the simulations mentioned above. In this simulation, firstly both hole and electron-transport layers are optimized with respect to the energy band gap, electron affinity, film thickness, and doping density and then current-voltage characteristics are simulated with ideal holes and electron-transport layer, and the result is shown in Figure 12. The final photocurrent-voltage characteristics display that the FACsSnI_3_-based perovskite solar cell with ideal electron and hole-transport layers works very well. Figure 11 demonstrates the photovoltaic parameters such as open-circuit voltage, short-circuit current, and fill factor and their values are found 1.3 eV, 24.0 mA·cm^−2^, and 77%, respectively. All these parameters lead to the maximum power-conversion efficiency of up to ~24% for FACsSnI_3_-based perovskite solar cells with an ideal hole and electron-transport layer.

## 4. Conclusions

Finally, it can be concluded that, after being properly designed, the recently reported FACsSnI_3_-based perovskite solar cell has the potential to replace lead-based perovskite solar cells. Extensive simulation and modeling with each design variable, including electron affinity, energy band gap, film thickness, and doping density of both electron and hole-transport layers, were carried out to find the most optimal parameters of both suitable transport layers. Observations indicate that an ideal hole-transport layer should have an electron affinity of 2.12 eV and an energy band gap of 2.0 eV, while an ideal electron-transport layer should have an electron affinity of 2.0 eV and an energy band gap of 4.0 eV. The thickness of FACsSnI_3_ perovskite is similarly optimized at 100 nm, yielding maximum open-circuit voltage, short-circuit current, and fill-factor values of around 1.3 eV, 24.0 mA·cm^−2^, and 77%, respectively. All of these improved parameters result in a maximum power-conversion efficiency of 24% for FACsSnI_3_-based perovskite solar cells with a perfect hole and electron-transport layer. We expect this study’s findings will serve as a reliable reference for future work on the study and development of lead-free perovskite solar cells.

## Figures and Tables

**Table 1:** Yearly milestone efficiency of FACsSnI_3_ (FA_0.85_Cs_0.15_SnI_3_) based perovskite solar cell with hole-transport layer (HTL), electron-transport layer (ETL), and power-conversion efficiency (PCE).

Year	Author	Perovskite	HTL	ETL	PCE (%)	Ref
2016	Lee et al.	FACsSnI_3_	Spiro-OMeTAD	TiO_2_	4.8	[31]
2016	Lio et al.	FACsSnI_3_	PEDOT:PSS	PCBM	6.07	[29]
2018	Kim et al.	FACsSnI_3_	PEDOT:PSS	BCP	7.66	[32]
2019	Ran et al.	FACsSnI_3_	PEG-PEDOT:PSS	C60	9.61	[33]
2020	Nie et al.	FACsSnI_3_	CuSCN	PCBM	7.34	[34]
2021	Yu et al.	FACsSnI_3_	PEG-PEDOT:PSS	C60	9.0	[35]
2021	Li et al.	FACsSnI_3_	PEDOT:PSS	C60	10.4	[30]
2022	Zillner et al.	FACsSnI_3_	NiOx	PCBM	6.76	[36]

**Table 3:** Optimized parameters of hole and electron-transport layers for FACsSnI_3_-based perovskite solar cell estimated from simulations.

Transport Layer	Electron Affinity	Energy Band Gap	Thickness	Doping Density
	(eV)	(eV)	(nm)	(/cm^3^)
Hole Transport	2.12	2.7	20	10^20^
Electron Transport	4.0	2.0	260	10^16^
FACsSnI_3_	-	-	100	-

## Data Availability

Available on request.
